# Physical Activity and Sedentary Behavior of Cancer Survivors and Non-Cancer Individuals: Results from a National Survey

**DOI:** 10.1371/journal.pone.0057598

**Published:** 2013-03-06

**Authors:** Roy B. Kim, Allison Phillips, Kirsten Herrick, Marieka Helou, Carlin Rafie, Mitchell S. Anscher, Ross B. Mikkelsen, Yi Ning

**Affiliations:** 1 Virginia Commonwealth University School of Medicine, Richmond, Virginia, United States of America; 2 Department of Epidemiology and Community Health, Virginia Commonwealth University School of Medicine, Richmond, Virginia, United States of America; 3 Department of Pediatric Oncology, Virginia Commonwealth University Medical Center, Richmond, Virginia, United States of America; 4 Massey Cancer Center, Virginia Commonwealth University Medical Center, Richmond, Virginia, United States of America; 5 Department of Radiation Oncology, Virginia Commonwealth University Medical Center, Richmond, Virginia, United States of America; Brigham and Women’s Hospital and Harvard Medical School, United States of America

## Abstract

Increasing physical activity and decreasing sedentary behavior are associated with a higher quality of life and lower mortality rates for cancer survivors, a growing population group. Studies detailing the behavior of cancer survivors are limited. Therefore, we investigated physical activity and sedentary behavior of cancer survivors using data from the National Health and Nutrition Examination Survey (NHANES) 2007–2010. Participants were those who provided physical activity and sedentary behavior data. Those who were pregnant, <20 years old, or <3 years from their cancer diagnosis were excluded. A cancer case was a self-reported diagnosis by a physician. We identified 741 cancer survivors and 10,472 non-cancer participants. After adjustment for age, race, gender, education status, body mass index, and smoking status, cancer survivors (n = 10,472) reported significantly longer duration of sedentary behavior (OR = 1.42, 95% CI (1.12, 1.80) for 8 or more hours, p-value for trend = 0.09), compared to non-cancer participants (n = 741). They also reported non-significant increases in maximum intensity, duration, frequency, and energy expenditure, whereas they reported significant increases in moderate intensity (OR = 1.26, 95% CI (1.01, 1.57)), moderate frequency (1–4 times/week) (OR = 1.32, 95% CI (1.00, 1.74)), and moderate energy expenditure (4018.5–7623.5 kcal) (OR = 1.30, 95% CI (1.00, 1.71)) of physical activity, compared to non-cancer participants. These patterns are similar for breast and prostate cancer survivors, with prostate cancer survivors more likely to engage in physical activity for more than one hour per day (OR = 1.98, 95% CI (1.05, 3.71)). Our findings suggest that cancer survivors tend to have more physical activity, but they are also more likely to engage in sedentary behavior.

## Introduction

In the United States, one in every four deaths is caused by cancer, and it is the leading cause of death for people under the age of eighty [1], [2]. It is estimated that 638,910 new cases will be diagnosed and 577,190 cancer-related deaths will occur in the United States in 2012 [2]. Overall cancer incidence is projected to increase by 75% worldwide by the year 2030 [3]. In addition, previous research has demonstrated that compared to individuals without a history of cancer, cancer survivors are significantly more likely to develop secondary complications, such as additional cancers, cardiovascular diseases, and a general decline in quality of life [1], [4], [5].

Despite the increases in cancer incidence, there is data to suggest that cancer mortality is on the decline. Between 1999 and 2008, mortality rates decreased 1% per year among both males and females for almost all ethnic groups [2]. The rate of decline appears to be accelerating; yearly decreases in cancer mortality rates between 2004 and 2008 were 1.8% and 1.6% annually for males and females, respectively [2]. As a result, with the number of cancer survivors continuing to grow, the study of this population group is becoming increasingly important.

Better diagnostic tools and advanced treatment options have clearly contributed to the decrease in cancer mortality rates [6]. Additionally, maintaining a healthy lifestyle is the most important strategy to improving post-diagnostic quality of life and decreasing mortality rates for most cancers [7], [8]. Certain lifestyle habits, such as smoking abstinence or cessation, exercise, and healthy diet, are associated with reduced cancer mortality [6]. Physical activity, arguably one of the most important lifestyle factors, has been studied extensively because it has been both linked to decreased mortality rates and has shown the strongest association to health related quality of life [6], [9]–[11]. Proposed mechanisms include cardiovascular fitness, muscular strength, less fatigue, and a general improvement in patient-rated physical functioning [12].

The increasing incidence of cancer and the decreasing mortality trends both suggest that we will have greater numbers of cancer survivors in the future. Thus, we believe it is very important to study the actual behavior of this ever-growing population. Previous research has studied various behavioral changes in patients following a cancer diagnosis [9], [13]–[14]. Our present study specifically examined the physical activity and sedentary behaviors of cancer survivors compared to non-cancer participants.

## Methods

### Study Population

The National Health and Nutrition Examination Survey (NHANES) for the years 2007–2010 was the primary source of data. Earlier cycles were not included because the physical activity questionnaire changed between the 05–06 and 07–08 cycles. NHANES is a program of studies conducted by the National Center of Health Statistics (NCHS) that is used to assess the health and nutritional statuses of both adults and children of all ethnicities living in the United States; it is a combination of interviews, physical exams, and validated questionnaires given to randomly selected participants [15].

The initial screened sample included 14,286 participants who completed the questionnaires. We then excluded participants if they were less than 20 years old, currently pregnant, had any major socio-demographic or clinical factors missing (smoking, cancer, education, BMI, physical activity/sedentary behavior), or were less than 3 years removed from their cancer diagnosis. We excluded those who were less than 3 years from their cancer diagnosis to eliminate individuals currently on treatment or having recently completed treatment, as this could affect the variables we were studying. Our final analytic population had 11,213 participants: 741 cancer survivors and 10,472 non-cancer participants.

### Assessment of Cancer

Cancer was the primary outcome of interest for the present study. Cancer survivors were defined as having cancer if they responded “Yes” to the interview question, “Have you ever been told by a doctor or other health professional that you had cancer or a malignancy of any kind?” Individuals with a non-melanoma cancer were not categorized as cancer survivors, unless they had an additional form of cancer. Thus, the final group of “cancer survivors” included patients that met the aforementioned qualifications of cancer and were at least 3 years removed from their cancer diagnoses.

### Assessment of Physical Activity and Sedentary Behavior

Detailed information of physical activity and sedentary behavior was assessed via a questionnaire-based interview. Questions regarding physical activity assessed frequency and duration from a “typical week” in the participant’s life. Moderate physical activity was described as an activity, during work or for recreation, which resulted in a small increase in breathing or heart rate and was done for at least ten minutes continuously [16]. Moderate physical activity also included any walking or biking the participant did while going to work or traveling from place to place [16]. A vigorous physical activity had all the same parameters, but was phrased as a “large” increase in breathing or heart rate [16]. Vigorous physical activity included carrying or lifting heavy loads, digging, or construction work [16]. Sedentary activity questions were phrased as the average time per day, over the last 30 days, spent sitting or reclining at work, home, or school; this included time spent sitting at a desk, with friends, in a car, bus, or train, reading, playing cards, watching television, or using a computer [16]. This variable did not include time spent sleeping [16].

The metabolic equivalent (MET) values were provided by NHANES to assess energy expenditure and were assigned based on whether the activity was considered moderate or vigorous. MET is defined as 1 MET = 1 kcal/(kg×hour) [15]. The MET values were multiplied by average duration and average times per week of physical activity to obtain MET min/week. These scores were then organized into tertiles.

### Covariates

Covariates were chosen *a priori* based on a literature search; we included age, sex, race/ethnicity, education, smoking status, alcohol consumption, and Body Mass Index (BMI). The type of cancer and duration (in years) since diagnosis at the time of the interview are reported for participants with cancer.

### Statistical Analysis

Sampling weights were used in all analyses to account for the complex sampling design. Demographic and health-related characteristics, such as age, race, gender, smoking status, and education, were compared by cancer status. A chi-square test or a t-test was used to determine if there was a significant difference. These characteristics are summarized for both cancer survivors and non-cancer participants with un-weighted sample sizes, weighted percentages, and p-values. Continuous variables are summarized with means and standard errors (SE).

Multivariable logistic regressions were used to test for relationships between physical activity, sedentary behavior factors, and cancer status. Results are presented as odds ratios (OR) and 95% confidence intervals (CI). Maximum intensity of all physical activity (none, moderate, vigorous), the duration of all moderate and vigorous physical activity (0, 1–60, >60 minutes per day), the frequency of all moderate and vigorous physical activity (0, 1–4, 5+ days per week), and energy expenditure of physical activity were studied for physical activity. Sedentary behavior was analyzed as duration per day (<5, 5 to <8, and >8 hours per day).

Two separate models were run; the first was adjusted for age and the second was adjusted for age, race/ethnicity (Non-Hispanic White, Non-Hispanic Black, Mexican-American, other), gender, education (secondary education (undergraduate level) or less, post-secondary), smoking status (never smoked, former smoker, current smoker), and BMI. Alcohol drinking was not included in the final models because it changed the ORs less than 10%. Subgroup analyses were performed for breast and prostate cancer survivors. The significance of linear trends across categories of physical activity and sedentary behavior was evaluated using a signed score for each category as a continuous variable. All statistical analyses were performed by using SAS statistical software (version 9.3; SAS Institute, Cary, NC).

## Results

Socio-demographic and clinical characteristics by cancer status are provided in Table 1. As expected, cancer survivors were older than non-cancer participants (61.9±0.71 vs. 45.7±0.31). Men made up 39.8% of the cancer group, while representing 49.1% of the non-cancer group. Cancer survivors were more likely to be non-Hispanic white and people who never smoked or used to smoke. The two groups showed no significant differences in BMI or postsecondary education percentage. The final cancer sample had 11 lung, 132 breast, 141 prostate, 79 colon, and 378 “other” cancers. The “other” cancer group includes less prevalent cancers or cases in which specific diagnosis information was not available.

**Table 1:** Demographic and health-related characteristics of study participants by cancer status in the NHANES 2007–2008 and 2009–2010 cycles*.

Characteristic	Cancer	Non-Cancer	P-value
Sample Size (n)	741	10472	<0.001
Age (y)	61.9±0.71	45.7±0.31	<0.001
Men (%)	39.8	49.1	0.002
Race (%)			<0.001
Non-Hispanic White	86.2	67.5	
Non-Hispanic Black	6.4	11.6	
Mexican American	2.6	8.8	
Other	4.8	11.9	
Education, postsecondary (%)	57.6	56.2	0.66
Smoking Status			0.015
Never/Former	82.7	77.9	
Current	17.3	22.1	
BMI (kg/m2)	29.0±0.29	28.6±0.10	0.21
Alcohol consumption			<0.001
Yes	395	6332	
No	93	1288	
Ever or missing	253	2852	
Type of Cancers			
Lung	11		
Breast	132		
Prostate	141		
Colon	79		
Other**	378		
Duration since diagnosis of cancer			
Less than 3 years	208		
3 to <5 years	103		
5+ years	638		
Maximum intensity of physical activity (%)			<0.001
None	34.1	28.7	
Moderate	42.6	31.8	
Vigorous	23.3	39.5	
Duration of all moderate and vigorous physical activity (%)			<0.001
0 minutes per day	34.4	28.8	
1 to 60 minutes per day	27.2	23	
>60 minutes per day	38.4	48.2	
Frequency of all moderate and vigorous physical activity (%)			0.003
0 days per week	34.4	28.7	
1 to 4 days per week	24.9	23.2	
5+ days per week	40.6	48.1	
Duration of sedentary behavior (%)			<0.001
<5 hours per day	36.6	46.5	
5 to <8 hours per day	28.6	24.7	
8+ hours per day	34.9	28.8	
Energy expenditure (METs/wk)			<0.001
Tertile 1 (≤2033.5)	34.4	28.8	
Tertile 2 (4081.5–7603.5)	37.3	34.1	
Tertile 3 (≥7693)	28.3	37.1	

* those with time since diagnosis <3 years excluded.

** Other cancer includes lesser prevalent cancers or the specific cancer information was not available.

### Physical Activity

The results of our main analysis are presented in Table 2, which provides maximum intensity of all physical activity, duration and frequency of all moderate and vigorous physical activity, duration of sedentary behavior, and energy expenditure of physical activity.

**Table 2:** Association of Physical Activity and Sedentary Behavior with Cancer in the NHANES 2007–2008 and 2009–2010 Cycles*.

Characteristic	No. of Cancer	No. of Non-cancer	OR (95% CI)^1^	OR (95% CI)^2^
Physical activity				
No	304	3762	Reference	Reference
Yes	437	6710	1.24 (1.00, 1.53)	1.17 (0.94, 1.46)
Maximum intensity of physical activity				
None	304	3762	Reference	Reference
Moderate	288	3168	1.36(1.09, 1.70)	1.26 (1.01, 1.57)
Vigorous	149	3452	1.04(0.80, 1.35)	1.01(0.75, 1.36)
*p for trend*			*0.97*	*0.41*
Duration of moderate and vigorous physical activity				
0 minutes per day	304	3762	Reference	Reference
1 to 60 minutes per day	170	2232	1.30 (0.98, 1.73)	1.28 (0.97, 1.70)
>60 minutes per day	267	4478	1.19 (0.96, 1.47)	1.24 (0.99, 1.56)
*p for trend*			*0.07*	*0.19*
Frequency of all moderate and vigorous physical activity				
0 days per week	304	3758	Reference	Reference
1 to 4 days per week	165	2227	1.32 (1.00, 1.74)	1.32 (1.00, 1.74)
5+ days per week	272	4487	1.19 (0.95, 1.48)	1.22 (0.96, 1.54)
*p for trend*			*0.04*	*0.39*
Energy expenditure (METs/wk)				
Tertile 1(≤2033.5)	304	3762	Reference	Reference
Tertile 2(4081.5–7623.5)	236	3176	1.31 (1.01, 1.71)	1.30 (1.00, 1.71)
Tertile 3(≥7693)	201	3534	1.14 (0.97, 1.43)	1.20 (0.96, 1.51)
*p for trend*			*0.52*	*0.17*
Duration of sedentary behavior				
<5 hours per day	307	5364	Reference	Reference
5 to <8 hours per day	215	2537	1.27 (1.01, 1.59)	1.22 (0.98, 1.53)
8+ hours per day	219	2571	1.48 (1.15, 1.91)	1.42 (1.12, 1.80)
*p for trend*			*0.012*	*0.09*

* those with time since diagnosis <3 years excluded.

1 adjusted by age.

2 adjusted for age, race, gender, education, Body Mass Index, smoking status.

Cancer survivors were more likely to report engaging in physical activity (multivariable adjusted OR = 1.17, 95% CI (0.94, 1.46)). Likewise, they were more likely to report moderate physical activity as their maximum intensity level, as opposed to survey participants without a history of cancer (multivariable adjusted OR = 1.26, 95% CI (1.01, 1.57)). No significant difference was observed for the vigorous maximum intensity variable (multivariable adjusted OR = 1.01, 95% CI (0.75, 1.36)).

The results indicated that cancer survivors reported marginally significant increases in duration and frequency of physical activity, although the associations were not linear (p-values for trend were 0.19 and 0.39 for duration and frequency respectively). For duration, the multivariable adjusted ORs for 1 to 60 minutes per day and >60 minutes per day were 1.28, 95% CI (0.97, 1.70) and 1.24, 95% CI (0.99, 1.56) respectively. For frequency, 1 to 4 days per week and >5 days per week showed multivariable adjusted ORs of 1.32, 95% CI (1.00, 1.74) and 1.22, 95% CI (0.96, 1.54) respectively.

Cancer survivors had a non-significantly higher level of energy expenditure (p-value for trend = 0.17) compared to non-cancer participants. Cancer survivors were more likely to be in tertile 2 vs. tertile 3 (multivariable adjusted ORs of 1.30, 95% CI (1.00, 1.71) vs. 1.20 (0.96, 1.51) respectively).

### Sedentary Behavior

Cancer survivors were more likely to report spending greater than 8 hours per day engaged in sedentary behavior (OR = 1.42, 95% CI (1.12, 1.80)) after adjustment for multiple confounding factors. They also reported a non-significant increase in sedentary behavior from 5 to 8 hours (OR = 1.22, 95% CI (0.98, 1.53) adjusted). Thus, the data suggests that cancer survivors had a greater probability to be inactive for longer periods of time compared to non-cancer participants (p-value for trend = 0.09).

### Physical Activity and Sedentary Behavior for Breast and Prostate Cancer Survivors

Results of subgroup analysis for breast and prostate cancer are presented in Table 3. Similar to all cancer survivors, breast cancer survivors significantly preferred a moderate intensity level of physical activity (OR = 1.74, 95% CI (1.07, 2.83)). They were also significantly more likely to report spending 1 to 60 minutes per day engaged in moderate and vigorous physical activity (OR = 1.80, 95% CI (1.10, 2.94)) and greater than 8 hours a day in sedentary behavior (OR = 1.99, 95% CI (1.25, 3.19)). In contrast, prostate cancer survivors were significantly more likely to spend greater than 60 minutes per day engaged in moderate and vigorous physical activity (multivariable adjusted OR = 1.98, 95% CI (1.05, 3.71)). A moderate level intensity of all physical activity (OR = 1.73, 95% CI (1.01, 2.96)) and a higher level of energy expenditure for physical activity were also shown to be significantly more likely (p-value for trend = 0.027) for prostate cancer survivors.

**Table 3:** Association of Physical Activity and Sedentary Behaviors with Breast and Prostate Cancer in the NHANES 2007–2008 and 2009–2010 Cycles*.

Characteristic	No. of Cancer	No. of Non-cancer	OR (95% CI)^1^	OR (95% CI)^2^
**Breast Cancer (n = 132)**				
Physical activity				
No	53	2249	Reference	Reference
Yes	79	3081	1.83 (1.15, 2.90)	1.59 (0.97, 2.60)
Maximum intensity of all physical activity				
None	53	2249	Reference	Reference
Moderate	64	1846	1.25 (0.62, 2.54)	1.74 (1.07, 2.83)
Vigorous	15	1235	1.02 (1.27, 3.21)	1.06 (0.49, 2.54)
*p for trend*			*0.29*	*0.87*
Duration of all moderate and vigorous physical activity				
0 minutes per day	53	2255	Reference	Reference
1 to 60 minutes per day	47	1305	2.10 (1.31, 3.39)	1.80 (1.10, 2.94)
>60 minutes per day	32	1769	1.55 (0.84, 2.87)	1.36 (0.69, 2.66)
*p for trend*			*0.93*	*0.62*
Frequency of all moderate and vigorous physical activity				
0 days per week	53	2250	Reference	Reference
1 to 4 days per week	35	1162	1.71 (0.99, 2.93)	1.48 (0.87, 2.53)
5+ days per week	44	1918	1.91 (1.14, 3.21)	1.66 (0.94, 2.95)
*p for trend*			*0.02*	*0.11*
Energy Expenditure (METs/wk)				
Tertile 1 (≤1154.5)	53	2255	Reference	Reference
Tertile 2 (2317.5–3842)	40	1280	1.75 (0.98, 3.10)	1.49 (0.83, 2.66)
Tertile 3 (≥3896.5)	39	1794	1.91 (1.11, 3.30)	1.68 (0.93, 3.04)
*p for trend*			*0.51*	*0.57*
Duration of sedentary behavior				
<5 hours per day	46	2696	Reference	Reference
5 to <8 hours per day	46	1300	1.61 (0.96, 2.70)	1.48 (0.88, 2.50)
8+ hours per day	40	1321	2.28 (1.43, 3.64)	1.99 (1.25, 3.19)
*p for trend*			*0.003*	*0.005*
**Prostate Cancer (n = 141)**				
Physical activity				
No	56	1516	Reference	Reference
Yes	85	3658	1.61 (1.01, 2.58)	1.57 (0.88, 2.79)
Maximum intensity of all physical activity				
None	56	1516	Reference	Reference
Moderate	56	1333	1.79 (1.13, 2.83)	1.73 (1.01, 2.96)
Vigorous	29	2325	1.32 (0.68, 2.55)	1.27 (0.58, 2.79)
*p for trend*			*0.75*	*0.94*
Duration of all moderate and vigorous physical activity				
0 minutes per day	56	1525	Reference	Reference
1 to 60 minutes per day	25	928	1.06 (0.53, 2.11)	1.03 (0.48, 2.19)
>60 minutes per day	60	2718	2.02 (1.21, 3.38)	1.98 (1.05, 3.71)
*p for trend*			*0.42*	*0.40*
Frequency of all moderate and vigorous physical activity				
0 days per week	56	1519	Reference	Reference
1 to 4 days per week	34	1065	1.87 (1.05, 3.23)	1.79 (0.96, 3.37)
5+ days per week	51	2590	1.47 (0.86, 2.53)	1.44 (0.74, 2.18)
*p for trend*			*0.30*	*0.55*
Energy expenditure (METs/wk)				
Tertile 1 (≤791)	57	1664	Reference	Reference
Tertile 2 (1586–3436)	56	1764	1.82 (1.13, 2.92)	1.78 (0.99, 3.20)
Tertile 3 (≥3460)	23	1743	1.48 (0.87, 2.51)	1.48 (0.81, 2.72)
p for trend			0.006	0.027
Duration of sedentary behavior				
<5 hours per day	63	2668	Reference	Reference
5 to <8 hours per day	36	1237	1.01 (0.63, 1.62)	1.02 (0.64, 1.61)
8+ hours per day	42	1250	1.24 (0.72, 2.14)	1.21 (0.70, 2.10)
*p for trend*			*0.43*	*0.43*

* those with time since diagnosis <3 years excluded.

1 adjusted by age.

2 adjusted for age, race, education, Body Mass Index, smoking status.

Sensitivity analysis, including cancer survivors that were less than three years from their cancer diagnosis, did not appreciably change the results.

## Discussion

The present study showed that cancer survivors were more likely to report engaging in physical activity, especially at the moderate intensity level, resulting in more physical activity energy expenditure compared to individuals without a history of cancer. However, they were also more likely to report spending greater than 8 hours a day in a sedentary state when compared to non-cancer participants. Results from subgroup analysis for breast and prostate cancer survivors were similar for sedentary behavior. Breast cancer survivors were more likely to report spending 1 to 60 minutes per day on all moderate and vigorous physical activity. Prostate cancer survivors were more likely to report spending >60 minutes per day on all moderate and vigorous physical activity while generally exerting more energy.

The significant differences in physical activity levels between cancer survivors and non-cancer participants suggest an intention to make positive lifestyle adjustments by cancer survivors in terms of exercise. Previous studies have demonstrated similar changes, with cancer survivors being 9% more likely to meet physical activity recommendations compared with non-cancer participants [9], [17]. In contrast, other studies found either no significant difference in age-stratified behavioral risk factor prevalence between cancer survivors and non-cancer participants or a cancer diagnosis having a negative influence on exercise [13]–[14]. Additionally, there is data showing low adherence to the American Cancer Society’s recommendation for physical activity for cancer survivors [18]. We believe that these results are more representative of the lack of physical activity in the general population as a whole rather than the lack of behavioral adjustment following a cancer diagnosis [9]. Regardless, there is a need for future studies to look at physical activity level changes in more detail.

Although they were relatively active overall, cancer survivors were significantly more likely to report spending greater than 8 hours in sedentary behavior compared to non-cancer participants. Past research supports this finding, as levels of physical inactivity were found to be higher in cancer survivors [19]. Coupling this with our significant finding of moderate exercise being more prominent in cancer survivors suggests that this population group prefers a less than vigorous level of physical activity when they do exercise, and have a much higher level of inactivity. One possible explanation for this behavior is the nearly universal symptom of fatigue seen in cancer patients [20]. Although more often seen in cancer patients undergoing cancer treatments and therapies, fatigue is still a major obstacle all cancer patients must face to achieve a higher quality of life [21]–[22]. Thus, fatigue may cause a moderate level of physical activity to become preferred over a vigorous level, and also further promotes sedentary behavior.

Similar to all cancer survivors, breast cancer survivors reported a higher incidence of spending greater than 8 hours a day in sedentary behavior. However, they were also more likely to spend between 1 to 60 minutes per day exercising either moderately or vigorously. Prostate cancer survivors were more likely to spend greater than 60 minutes per day on moderate or vigorous exercise. Although both male and female cancer survivors are equally likely to be involved in physical activity, males were significantly more likely to engage in the vigorous intensity level [23].

Post-hoc analysis including cancer survivors who were within 3 years of diagnosis did not significantly alter the results. Other studies have also demonstrated that physical activity levels did not depend the on time since diagnosis [9]. Thus, this implies that there is an adjustment in behavior immediately following diagnosis without subsequent changes in physical activity and sedentary behavior. Rather, any changes in lifestyle behavior seem to be relatively stable. This suggests that the diagnosis of cancer creates a great potential for a “teachable moment” and that cancer patients could possibly be more receptive to change [13], [24].

Although this study provides numerous strengths, one being representative of existing cancer survivors in the U.S., there are some limitations that should be considered. One limitation is that the cancer diagnosis was self-reported by each participant; participants were instructed to only report a physician’s diagnosis of cancer. However, we expect any reporting errors to be minimal because cancer is a life threatening disease. Another limitation is the lack of information on cancer stage. The stage of cancer has been shown to influence one’s motivation and energy levels, and therefore should be considered when looking at behavioral patterns [25]. Finally, physical activity and sedentary behavior were self-reported. Nonetheless, accurate measures of physical activity are always a challenge in epidemiologic studies and we expect any errors to be random, which might dilute the reported associations.

In summary, our results suggest that cancer survivors tend to be more physically active in terms of duration, frequency and energy expenditure, but they are also more susceptible to a sedentary lifestyle. These patterns are similar for breast and prostate cancer survivors, with prostate survivors more likely to engage in physical activity for longer periods of time.
